# Cyclic stretch enhances the expression of Toll-like Receptor 4 gene in cultured cardiomyocytes via p38 MAP kinase and NF-κB pathway

**DOI:** 10.1186/1423-0127-17-15

**Published:** 2010-03-05

**Authors:** Kou-Gi Shyu, Bao-Wei Wang, Chiu-Mei Lin, Hang Chang

**Affiliations:** 1 Division of Cardiology, Shin Kong Wu Ho-Su Memorial Hospital, Taipei, Taiwan; 2 Graduate Institute of Medical Sciences, College of Medicine, Taipei Medical University, Taipei, Taiwan; 3 School of Medicine, Fu-Jen Catholic University, Taipei, Taiwan; 4 Department of Emergency Medicine, Shin Kong Wu Ho-Su Memorial Hospital, Taipei, Taiwan

## Abstract

**Background:**

Toll-like receptor 4 (TLR4) plays an important role in innate immunity. The role of TLR4 in stretched cardiomyocytes is not known. We sought to investigate whether mechanical stretch could regulate TLR4 expression, as well as the possible molecular mechanisms and signal pathways mediating the expression of TLR4 by cyclic mechanical stretch in cardiomyocytes.

**Methods:**

Neonatal Wistar rat cardiomyocytes grown on a flexible membrane base were stretched by vacuum to 20% of maximum elongation at 60 cycles/min. Western blot, real-time polymerase chain reaction, and promoter activity assay were performed. In vitro monocyte adhesion to stretched myocyte was detected.

**Results:**

Cyclic stretch significantly increased TLR4 protein and mRNA expression after 2 h to 24 h of stretch. Addition of SB203580, TNF-α antibody, and p38α MAP kinase siRNA 30 min before stretch inhibited the induction of TLR4 protein. Cyclic stretch increased, while SB203580 abolished the phosphorylated p38 protein. Gel shifting assay showed significant increase of DNA-protein binding activity of NF-κB after stretch and SB203580 abolished the DNA-protein binding activity induced by cyclic stretch. DNA-binding complexes induced by cyclic stretch could be supershifted by p65 monoclonal antibody. Cyclic stretch increased TLR4 promoter activity while SB203580 and NF-κB siRNA decreased TLR4 promoter activity. Cyclic stretch increased adhesion of monocyte to cardiomyocytes while SB203580, TNF-α antibody, and TLR4 siRNA attenuated the adherence of monocyte. TNF-α and Ang II significantly increased TLR4 protein expression. Addition of losartan, TNF-α antibody, or p38α siRNA 30 min before Ang II and TNF-α stimulation significantly blocked the increase of TLR4 protein by AngII and TNF-α.

**Conclusions:**

Cyclic mechanical stretch enhances TLR4 expression in cultured rat neonatal cardiomyocytes. The stretch-induced TLR4 is mediated through activation of p38 MAP kinase and NF-κB pathways. TLR4 up-regulation by cyclic stretch increases monocyte adherence.

## Introduction

Toll-like receptors (TLRs) are pattern recognition receptors that play an important role in the induction of innate immunity by recognition of exogenous pathogen-associated molecular patterns and endogenous ligands [1]. Innate immune and inflammatory pathways have been implicated in cardiac dysfunction after global myocardial ischemia [2]. TLR4, a member of the TLR family, is expressed on the cell surface of cardiac cells, including cardiomyocytes, smooth muscle cells, and endothelial cells. Increased TLR4 expression has been observed in cardiomyocytes from human and animals with heart failure [3]. TLR4 can modulate myocyte contractility, myocardial ischemia-reperfusion injury [4,5]. TLR4 also plays a role in myocardial dysfunction during bacterial sepsis [6,7], pressure overload induced cardiac hypertrophy [8], and doxorubicin-induced cardiomyopathy [9].

Cardiac myocytes have been reported to express functional TLR4 in lipopolysaccharide-treated myocytes, which can produce tumor necrosis factor-α (TNF-α) [10] and activate NF-κB [11]. Raised TNF-α production has been reported in chronic heart failure [12]. However, the direct effect of TNF-α on TLR4 in cardiac myocytes is not known.

Chronic heart failure is a state of chronic inflammation [13]. Therefore, the importance of a functionally intact innate immune system in the heart should be emphasized. Mechanical stress overload is able to induce inflammatory mediators and causes ventricular hypertrophy [14]. The cyclic strain system subjects cultured cells to repetitive stretching-relaxation at rates comparable to dynamic stretch overload in vivo. This system has been applied widely to study the molecular mechanisms of gene expression and signal transduction in many cell types [15-17]. To date, it is not reported yet whether mechanical stretch can induce expression of TLR4 in cardiomyocytes. Thus, we sought to investigate whether stretch could regulate TLR4 expression, as well as the possible molecular mechanisms and signal pathways mediating the expression of TLR4 by cyclic mechanical stretch in cardiomyocytes.

## Methods

### Primary cardiomyocyte culture

Cardiomyocytes were obtained from Wistar rats aged 2-3 days old by trypsinization, as previously described [17]. Cultured myocytes thus obtained were >95% pure as revealed by observation of contractile characteristics with a light microscope and stained with anti-desmin antibody (Dako Cytomation, Glostrup, Denmark). Cardiomyocytes were seeded on flexible membranes base of 6 culture wells at a cell density of 1.6 × 10^6 ^cells/well in Ham's F-10 containing 10% horse serum and 10% fetal calf serum. After 2 days in culture, cells were transferred to serum-free medium (Ham's F-12: DMEM; 1:1) and maintained for another 2 days. The enriched myocytes were then subjected to cyclic stretch. The study conforms to *Guide for the Care and Use of Laboratory Animals *published by the US National Institutes of Health (NIH Publication No. 85-23, revised 1996). The study was reviewed and approved by the Institutional Animal Care and Use Committee of the Shin Kong Wu Ho-Su Memorial Hospital.

### In vitro cyclic stretch on cultured cardiomyocytes

The Flexcell FX-2000 strain unit consists of a vacuum unit linked to a valve controlled by a computer program. Cardiomyocytes cultured on the flexible membrane base were subjected to cyclic stretch produced by this computer-controlled application of sinusoidal negative pressure with a peak level of ≅ 15 kPa at a frequency of 1 Hz (60 cycles per min) for various periods of time. To determine the roles of c-Jun N-terminal kinase (JNK), p38 MAP kinase or p42/p44 MAP kinase in the expression of stretch-induced TLR4 expression, cardiomyocytes were pretreated with SP600125 (20 μM, CALBIOCHEM^®^, San Diego, CA, USA), SB203580 (3 μM, CALBIOCHEM^®^), or PD98059 (50 μM, CALBIOCHEM^®^) for 30 min, respectively, followed by cyclic stretch. SP600125 is a potent, cell-permeable, selective, and reversible inhibitor of JNK. SB203580 is a highly specific, cell permeable inhibitor of p38 kinase. PD98059 is a specific and potent inhibitor of p42/p44 MAP kinase. For evaluation of secreted TNF-α on TLR4 expression, conditioned medium from stretched cardiomyocytes and exogenous addition of TNF-α (100 pg/mL, R&D Systems, Minneapolis, MN, USA) or angiotensin II (Ang II) were used to check the TLR4 protein expression by Western blot.

### Western blot analysis

Western blot was performed as previously described [16]. Anti-human TLR4 human polyclonal antibody, anti-rat TNF-α antibody, anti-rat TNF-α receptor antibody (a neutralizing antibody, R&D Systems), polyclonal anti-p38 MAP kinase and monoclonal anti-phospho p38 MAP kinase antibodies (Cell Signaling, Beverly, MA, USA), anti-mouse monoclonal NF-κB p65 antibody (Santa Cruz Biotechnology, Inc., CA, USA), and polyclonal anti-phospho-NF-κB p65 antibody (Cell Signaling) were used. Signals were visualized by chemiluminenescent detection. Equal protein loading of the samples was further verified by staining polyclonal antibody GAPDH (LabFrontier, Seoul, Korea) or C23 monoclonal antibody (Santa Cruz Biotechnology). All Western blots were quantified using densitometry.

### RNA isolation and reverse transcription

Total RNA was isolated from cells using the single-step acid guanidinium thiocyanate/phenol/chloroform extraction method. Total RNA (1 μg) was incubated with 200 U of m Moloney-Murine Leukemia Virus reverse transcriptase in a buffer containing a final concentration of 50 mmol/L Tris-Cl (pH 8.3), 75 mmol/L KCl, 3 mmol/MgCl_2_, 20 U of RNase inhibitor, 1 μmol/L polydT oligomer, and 0.5 mmol/L of each dNTP in a final volume of 20 μL. The reaction mixture was incubated at 42°C for 1 h and then at 94°C for 5 min to inactivate the enzyme. A total of 80 μL of diethyl pyrocarbonate treated water was added to the reaction mixture before storage at -70°C.

### Real-time Quantitative PCR

A Lightcycler (Roche Diagnostics, Mannheim, Germany) was used for real-time PCR. The primer used for TLR4 was: forward, 5'-GGGTGAGAAACGAGCT-3'; reverse, 5'-TTGTCCTCCCACTCGA-3'. GAPDH: forward, 5'-CATCACCATCTTCCAGGAGC-3'; reverse, 5'-GGATGATGTTCTGGGCTGCC-3'. Real-time RT-PCR was performed as described previously [15]. Individual PCR products were analyzed for DNA sequence to confirm the purity of the product.

### RNA interference

Cells were transfected with 800 ng TLR4, p38, or NF-κB annealed siRNA (Thermo Scientific, Waltham, MA, USA). TLR4 sense and antisense of siRNA sequences were, 5'-GAAAUGCCAUGAGCUUUAGUU-3' and 5'-PCUAAAGCUCAUGGCAUUUCUU-3', respectively. P38α sense and antisense of siRNA sequences were 5'-GUCAUCGGUAAGCUUCUGACUU-3' and 5'-PUCAUCGGUAAGCUUCUGACUU-3', respectively. NF-κB sense and antisense of siRNA sequences were 5'-GGACGUGUUGCAUAUUUAAUU-3' and 5'-PUUAAAUAUGCAACACGUCCUU-3', respectively. TLR4, p38, or NF-κB siRNA is a target-specific 20-25 nt siRNA designed to knockdown gene expression. For negative control, a nontargeting siRNA (scrambled siRNA) purchased from Dharmacon Inc. was used. Cardiomyocytes were transfected with siRNA oligonucleotides using Effectene Transfection Reagent as suggested by the manufacture (Qiagen Inc, Valencia, CA, USA). After incubation at 37°C for 24 h, cells were used for stretch, and subjected to analysis of Western blot.

### Electrophoretic mobility shift assay (EMSA)

Nuclear protein concentrations from cardiomyocytes were determined by Biorad protein assay. Consensus and control oligonucleotides (Research Biolabs, Singapore) were labeled by polynucleotides kinase incorporation of [γ32P]-ATP. Oligonucleotides sequences included NF-κB consensus 5'-AGTTGAGGGGACTTTCCCAGGC-3'. The NF-κB mutant oligonucleotides sequences were 5'-AGTTGAGGCGACTTTCCCAGG-3'. After the oligonucleotide was radiolabeled, the nuclear extracts (4 μg of protein in 2 μl of nuclear extract) were mixed with 20 pmol of the appropriate [γ32P]-ATP-labeled consensus or mutant oligonucleotide in a total volume of 20 μl for 30 min at room temperature. The samples were then resolved on a 4% polyacrylamide gel. Gels were dried and imaged by autoradiography. Controls were performed in each case with mutant oligonucleotides or cold oligonucleotides to compete with labeled sequences.

### Promoter activity assay

A -591 to +49 bp rat TLR4 promoter construct was generated as follows. Rat genomic DNA was amplified with forward primer, ACGCGTCCCCATGAACAAAC and reverse primer, AGATCTGGAACAATGCCATG. The amplified product was digested with MluI and BglII restriction enzymes and ligated into pGL3-basic luciferase plasmid vector (Promega Corp., Madison, WI, USA) digested with the same enzymes. For the mutant, the NF-γB binding sites were mutated using the mutagenesis kit (Stratagene, La Jolla, CA). Site-specific mutations were confirmed by DNA sequencing. Plasmids were transfected into cardiomyocytes using a low pressure-accelerated gene gun (Bioware Technologies, Taipei, Taiwan) essentially following the protocol from the manufacturer. Test plasmid at 2 μg and control plasmid (pGL4-Renilla luciferase) 0.02 μg was cotransfected with gene gun in each well, and then replaced by normal culture medium. Following stretch treatment, cell extracts were prepared using Dual-Luciferase Reporter Assay System (Promega) and measured for dual luciferase activity by luminometer (Turner Designs, Sunnyvale, CA, USA).

### In vitro monocyte adhesion assay

For monocyte labeling, the human monocytic cell line THP-1 (American Type Culture Collection, Rockville, MD, USA) were suspended in phosphate-buffered saline (1 × 10^6^/ml) containing 1 μM calcein-AM (Invitrogen Inc., Eugene, OR, USA) and incubated for 15 min at 37°C. Labeled THP-1 cells were washed twice with phosphate-buffered saline and suspended in Hanks' buffered salt solution then added (5 × 10^5^/ml) to monolayers of stretched cardiomyocytes. After incubation and gentle rotation for 60 min, washed with Hanks' buffered salt solution to remove unbound cells, the number of binding monocytes was counted under fluorescent microscopy.

### Measurement of tumor necrosis factor-α and angiotensin II concentration

Conditioned media from cardiomyocytes subjected to stretch and those from control (without stretch) cells were collected for TNF-α and Ang II measurement. The level of TNF-α was measured by a quantitative sandwich enzyme immunoassay technique (R&D Systems). The level of Ang II was measured by a quantitative sandwich enzyme immunoassay technique (Phoenix Pharmaceutical, Inc., Belmont, CA, USA). The lower limit of detection of TNF-α and Ang II was 5.8 pg/mL and 0.07 ng/mL, respectively.

### Statistical analysis

The data were expressed as mean ± SD. Statistical significance was performed with analysis of variance (GraphPad Software Inc., San Diego, CA, USA). The Dunnett's test was used to compare multiple groups to a single control group. Tukey-Kramer comparison test was used for pairwise comparisons between multiple groups after the ANOVA. A value of P < 0.05 was considered to denote statistical significance.

## Results

### Cyclic stretch enhances toll-like receptor 4 protein and mRNA expression in cultured cardiomyocytes

**Figure 1:**
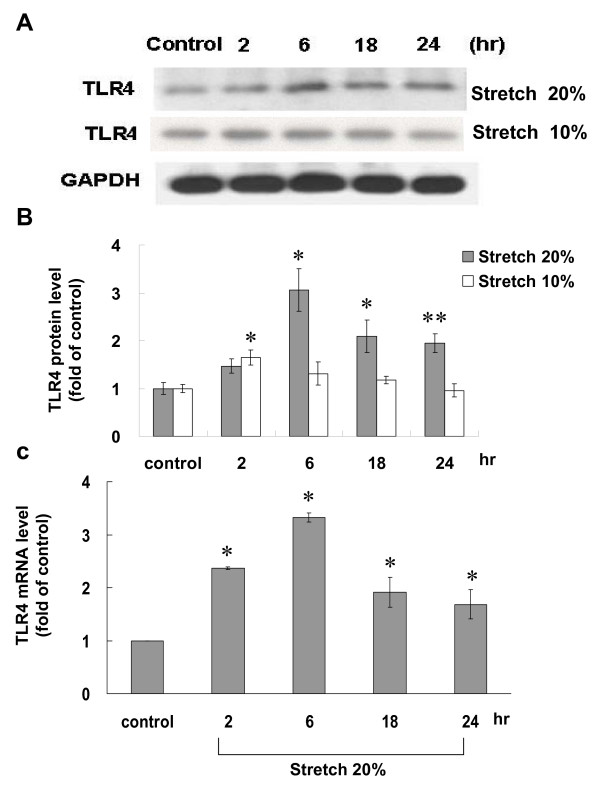
**Cyclic stretch increases toll-like receptor 4 (TLR4) protein and mRNA expression in cardiomyocytes**. (A) Representative Western blots for TLR4 in cardiomyocytes subjected to cyclic stretch by 20% or 10% for various periods of time. (B) Quantitative analysis of TLR4 protein levels. The values from stretched cardiomyocytes have been normalized to values in control cells and the data are from 4 independent experiments. *P < 0.001 vs. control. **P < 0.01 vs. control. (n = 4 per group) (C) Fold increases in TLR4 mRNA as a result of cyclic stretch by 20% for various periods of time. The values from stretched cardiomyocytes have been normalized to matched GAPDH measurement and then expressed as a ratio of normalized values to mRNA in control cells (n = 4 per group). *P < 0.01 vs. control.

To test the effect of cyclic stretch on the TLR4 expression, 10% and 20% of cyclic stretch were used. The levels of TLR4 protein began to increase as early as 2 h (1.5-fold) after stretch at 20% elongation was applied, reached a maximum of 3.1-fold over the control by 6 h and maintained elevated up to 24 h (Fig. 1). Stretch-induced TLR4 protein expression was load-dependent. When cardiomyocytes were stretched at 10% elongation, the levels of TLR4 protein slightly increased after stretch for 2 h and did not increase significantly as compared to control cells without stretch from 2 to 24 h (Fig. 1A and 1B). The levels of TLR4 mRNA also significantly increased from 2 h to 24 h after 20% of cyclic stretch (Fig. 1C). As shown in Additional file 1, cyclic stretch for 2 to 6 h also increased Ang II and TNF-α receptors protein expression in cardiomyocytes. This finding indicates that cyclic stretch could induce activations of Ang II and TNF-α receptors in cardiomyocytes.

### Cyclic stretch-induced TLR4 protein expression in cardiomyocytes is mediated by p38 MAP kinase and TNF-α

**Figure 2:**
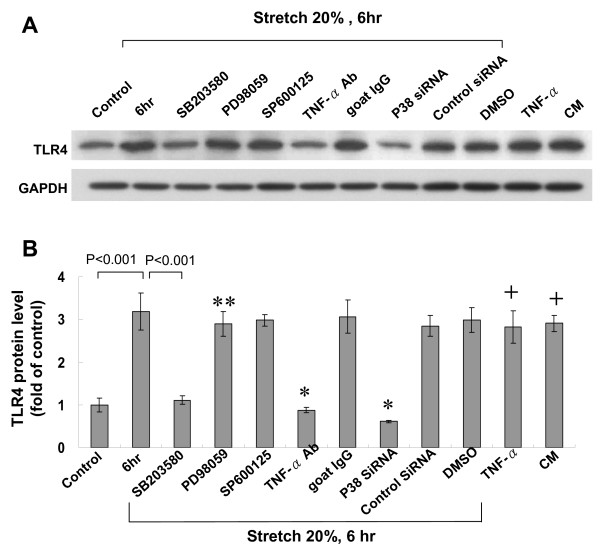
**p38 MAP kinase and tumor necrosis factor-α (TNF-α) are important regulators that mediate stretch-induced TLR4 expression in cardiomyocytes**. (A) Representative Western blots for TLR4 protein levels in cardiomyocytes subjected to cyclic stretch for 6 h or control cells without stretch in the absence or presence of different inhibitors, and siRNA. CM indicates conditioned medium. (B) Quantitative analysis of TLR4 protein levels. The values from stretched cardiomyocytes have been normalized to values in control cells (n = 4 per group). *P < 0.001 vs. stretch 6 h. **P < 0.01 vs. stretch 6 h. ^+^P < 0.001 vs. control.

**Figure 3:**
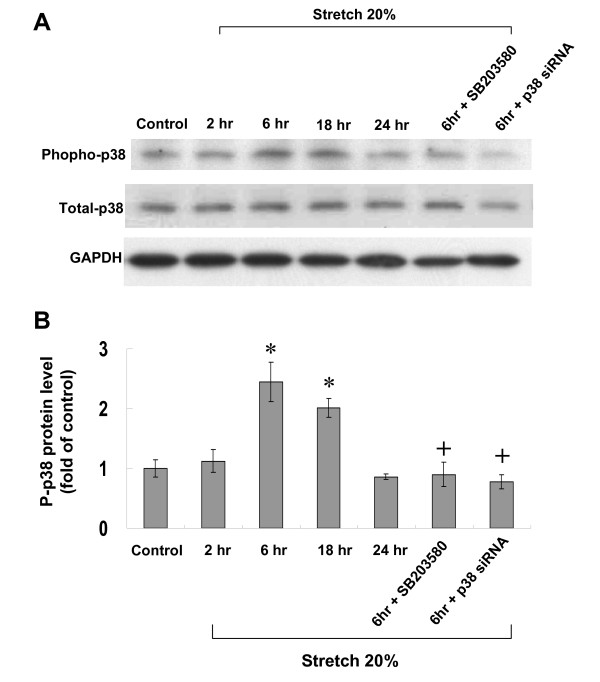
**Effect of cyclic stretch on expression of p38 kinase in cardiomyocytes**. (A) Representative Western blots for phosphorylated and total p38 kinases in cardiomyocytes after stretch by 20% for various periods of time and in the presence of SB203580 and p38 siRNA. (B) Quantitative analysis of phosphorylated p38 protein levels. The values from stretched cardiomyocytes have been normalized to matched GAPDH and corresponding total protein measurement and then expressed as a ratio of normalized values to each phosphorylated protein in control cells. Data are from 4 independent experiments. *P < 0.001 vs. control. ^+^P < 0.001 vs. stretch 6 h.

To investigate the possible signaling pathways mediating the stretch-induced TLR4 expression, different inhibitors were used. As shown in Fig. 2, the Western blots demonstrated that cyclic stretch-induced increase of TLR4 protein was significantly attenuated after addition of SB203580 before stretch. The TLR4 protein induced by stretch was partially attenuated by the addition of PD98059. DMSO as the vehicle for PD98059 did not affect TLR4 expression induced by stretch. P38α siRNA completely blocked the TLR4 expression induced by stretch. The control siRNA did not affect the TLR4 expression induced by stretch. As shown in Fig. 3, phosphorylated p38 protein was maximally induced at 6 h after cyclic stretch and remained elevated until 18 h. The phosphorylated p38 protein was abolished by pretreatment with SB203580. P38 siRNA knocked down the p38 protein expression by 67% (from 2.4-fold to 0.8-fold). These findings indicate that p38 MAP kinase pathway is an important regulator to mediate the TLR4 expression induced by cyclic stretch in cardiomyocytes.

**Figure 4:**
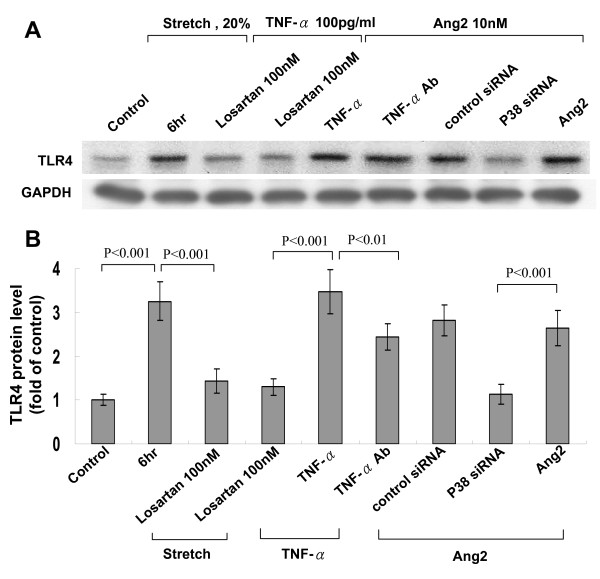
**Angiotensin II mediates the increase of TLR4 by cyclic stretch through angiotensin II receptor**. (A) Representative Western blots for TLR4 in cardiomyocytes subjected to cyclic stretch by 20% for 6 h or without stretch in the presence or absence of inhibitors. (B) Quantitative analysis of TLR4 protein levels. The values from stretched cardiomyocytes have been normalized to values in control cells and the data are from 4 independent experiments.

**Figure 5:**
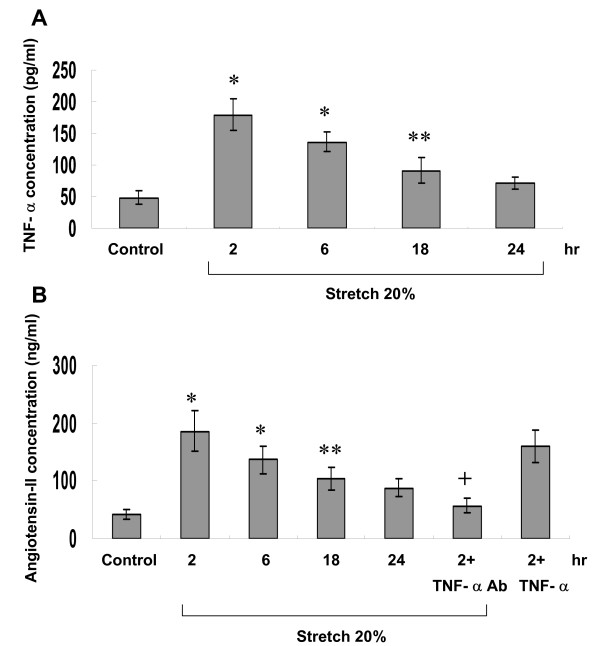
**Cyclic stretch increases release of TNF-α and angiotensin II (AngII) from cardiomyocytes subjected to 20% of stretch for various periods of time**. The cultured medium were collected for measurement of TNF-α (A) and Ang II (B) in cultured cardiomyocytes after stretch for various periods of time via immunoassay (n = 4). *P < 0.001 vs. control. **P < 0.05 vs. control. ^+^P < 0.001 vs. stretch 2 h.

**Figure 6:**
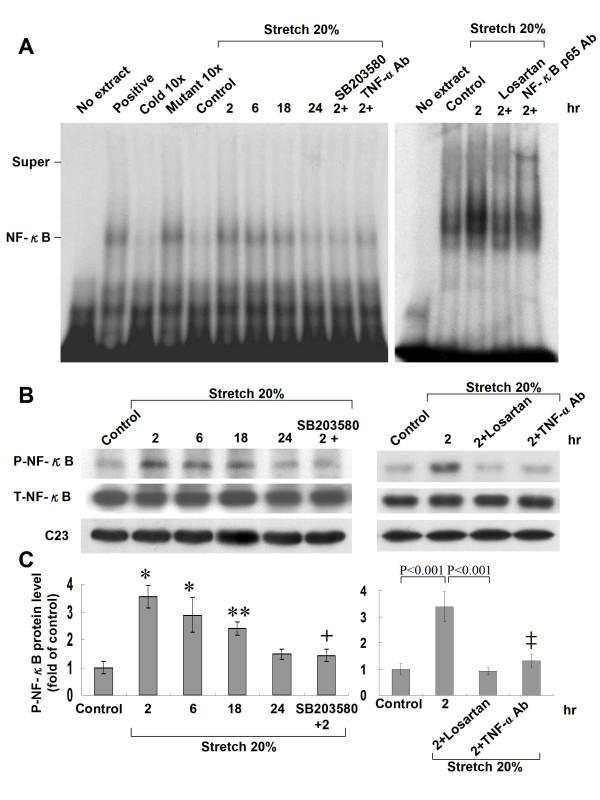
**Cyclic stretch increases NF-κB-binding activity and NF-κB protein phosphorylation**. (A) Representative EMSA showing protein binding to the NF-κB oligonucleotide in nuclear extracts of cardiomyocytes after cyclic stretch for various periods of time and in the presence of inhibitors. Similar results were found in another two independent experiments. Cold oligo means unlabeled NF-κB oligonucleotides. A supershifted complex is observed after addition of p65 antibody. (B) Representative Western blots for phosphorylated and total NF-κB in cardiomyocytes after stretch by 20% for various periods of time and in the presence of SB203580, losartan, and TNF-α antibody. (C) Quantitative analysis of phosphorylated NF-κB protein levels. The values from stretched cardiomyocytes have been normalized to matched C23 and corresponding total protein measurement and then expressed as a ratio of normalized values to each phosphorylated protein in control cells. Data are from 4 independent experiments. *P < 0.001 vs. control. **P < 0.01 vs. control. ^+^P < 0.001 vs. stretch 2 h. ^‡ ^P < 0.001 vs. stretch 2 h.

Exogenous addition of TNF-α at 100 pg/mL significantly increased TLR4 protein expression as compared to control cells (Fig. 2). Conditioned medium from stretched cardiomyocytes also significantly induced TLR4 protein expression. Addition of TNF-α antibody (5 μg/mL) 30 min before cyclic stretch completely inhibited the increase of TLR4 induced by cyclic stretch. Addition of goat IgG 30 min before cyclic stretch did not affect the protein expression of TLR4 induced by cyclic stretch. Addition of TNF-α receptor antibody (5 μg/mL) 30 min before stretch also significantly attenuated the increase of TLR4 induced by stretch (data not shown). This finding indicates that TNF-α may directly mediate the increase of TLR4 by cyclic stretch. As shown in Fig. 4, addition of losartan (100 nM) before stretch significantly inhibited the increase of TLR4 protein expression induced by stretch. Addition of losartan (100 nM) before TNF-α use significantly inhibited the increase of TLR4 protein expression induced TNF-α. Exogenous addition of Ang II significantly induced TLR4 protein expression. Addition of TNF-α antibody 30 min before addition of Ang II did not affect the TLR4 protein expression. Cyclic stretch significantly increased the TNF-α secretion from myocytes from 2 h to 18 h after stretch (Fig. 5A). The mean concentration of TNF-α rose from 47.9 ± 10.5 pg/mL before stretch to 179 ± 25 pg/mL after stretch for 2 h (P < 0.01). Cyclic stretch also significantly increased Ang II secretion from cardiomyocytes from 2 h to 24 h after stretch (Fig. 5B). The mean concentration of Ang II rose from 41.7 ± 8.4 ng/mL before stretch to 185.9 ± 34.9 ng/mL after stretch for 2 h (P < 0.01). Addition of TNF-α increased Ang II secretion from cardiomyocytes, while addition of TNF-α antibody significantly inhibited the Ang II secretion from stretched cardiomyocytes. These data indicate that cyclic stretch increases TLR4 protein expression through angiotensin receptor by Ang II and Ang II is secreted from cardiomyocytes by TNF-α stimulation. P38 siRNA attenuated the TLR4 expression induced by exogenous addition of Ang II, indicating Ang II receptor was activated before p38 MAP kinase. Combined these findings, our data indicate that cyclic stretch first increases TNF-α expression, then stimulates Ang II expression, which subsequently activates p38 MAP kinase and induces phosphorylation of NF-κB (Fig. 6) in cardiomyocytes.

### Cyclic stretch increases NF-κB-binding activity and NF-κB phosphorylation

Cyclic stretch of cultured cardiomyocytes for 2 to 24 h significantly increased the DNA-protein binding activity of NF-κB (Fig. 6A). An excess of unlabeled NF-κB oligonucleotide competed with the probe for binding NF-κB protein, whereas an oligonucleotide containing a 2-bp substitution in the NF-κB binding site did not compete for binding. Addition of SB203580, Ang II receptor antagonist with losartan, and TNF-α antibody 30 min before stretch abolished the DNA-protein binding activity induced by stretch (Fig. 6A). Our finding indicates that Ang II receptor-related mechanism is involved in cyclic stretch-induced NF-κB activity. DNA-binding complexes induced by cyclic stretch could be supershifted by a specific p65 antibody (a specific antibody for NF-κB), indicating the presence of this protein in these complexes. Cyclic stretch significantly increased phosphorylation of NF-κB as compared to control cells without stretch (Fig. 6B and 6C). The increased phosphorylation of NF-κB induced by stretch was significantly attenuated by addition of SB203580 30 min before stretch. Addition of losartan or TNF-α receptor antibody 30 min before stretch abolished the phosphorylation of NF-κB induced by stretch (Fig. 6B and 6C), indicating Ang II and TNF-α receptors are involved in cyclic stretch-induced NF-κB phosphorylation.

### Cyclic stretch increases TLR4 promoter activity

**Figure 7:**
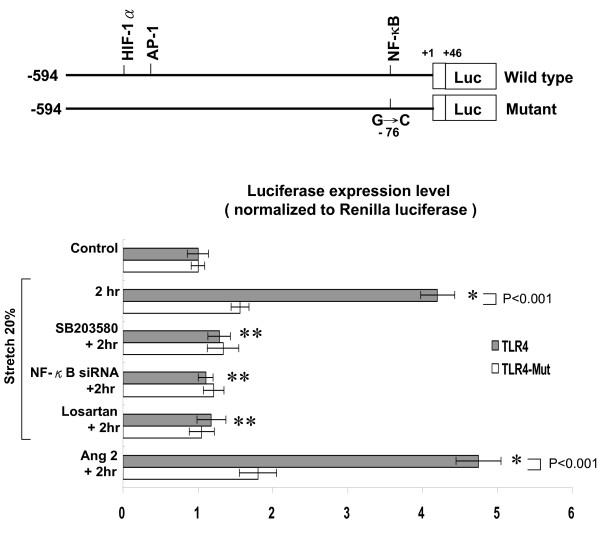
**Cyclic stretch increases TLR4 promoter activity**. Upper panel, constructs of TLR4 promoter gene. Lower panel, quantitative analysis of TLR4 promoter activity. Cardiomyocytes were transiently transfected with pTLR4-Luc by gene gun. The luciferase activity in cell lysates was measured and was normalized with renilla activity (n = 3 per group). *P < 0.001 vs. control. **P < 0.001 vs. 2 h.

The rat TLR4 promoter construct contains HIF-1α, AP-1, and NF-κB binding sites. Cyclic stretch for 2 h significantly increased the TLR4 promoter activity by 4.2-fold as compared to control without stretch (Fig. 7). Addition of SB203580 and NF-κB siRNA, losartan 30 minutes before stretch abolished the increased TLR4 promoter. When the NF-κB binding sites were mutated, the increased promoter activity induced by cyclic stretch was abolished. Exogenous addition of AngII increased the TLR4 promoter activity similar to cyclic stretch. This finding indicates that cyclic stretch regulates TLR4 in cardiomyocytes at transcriptional level and that NF-κB binding site in the TLR4 promoter is essential for the transcriptional regulation.

### Cyclic stretch increases monocyte adhesion

**Figure 8:**
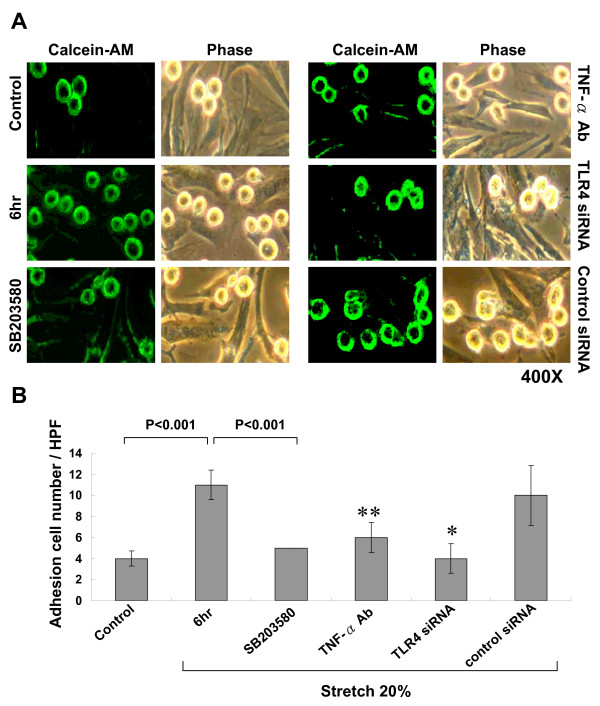
**Cyclic stretch increases adhesion of monocyte to stretched cardiomyocytes**. A, Representative microscopic image for monocyte adhesion assay with (left panel) or without green fluorescence (right panel) in cardiomyocytes subjected to cyclic stretch for 6 h or control cells without stretch in the absence or presence of inhibitors. B, Quantitative analysis of the positive fluorescent cells. (n = 4 per group). *P < 0.001 vs. 6 hr. **P < 0.01 vs. 6 hr

To test the function of increased TLR4 expression after cyclic stretch in cardiomyocytes, we performed monocyte adhesion assay. Monocyte adhesion to cardiomyocytes significantly increased after 6 h of stretch (2.7-fold) as compared to control cells without stretch (Fig. 8). Addition of SB203580, TNF-α antibody, and TLR-4 siRNA 30 min before stretch significantly attenuated the adhesion of monocyte to cardiomyocytes induced by stretch.

## Discussion

In this study, we demonstrated several significant findings. First, cyclic stretch up-regulates TLR4 expression in cardiomyocytes; second, TNF-α and AngII act as an autocrine factor to mediate the increased TLR4 expression induced by cyclic stretch; third, p38 MAP kinase and NF-κB transcription factor are involved in the signaling pathway of TLR4 induction, fourth, the increased TLR4 by stretch increases monocyte adhesion to cardiomyocytes. TLR4 in cardiomyocytes was up-regulated in both a time- and load-dependent manner by cyclic stretch. Our data clearly indicate that hemodynamic forces play a crucial role in the modulation of TLR4 expression in cardiomyocytes. Our data also demonstrated that functional consequence of TLR4 up-regulation by stretch resulted in adhesion of monocytes. TLR4 may activate monocyte activation to play host defense function [18]. However, the activated monocytes may decrease contractility of cardiomyocytes [19,20]. Left ventricular end-diastolic pressure is elevated in most of the diseased heart. The elevated end-diastolic pressure will stretch the myocardium. Therefore, the adherent monocytes to cardiomyocytes induced by stretch may worsen the ventricular function in diseased heart.

The induction of TLR4 protein by cyclic stretch was largely mediated by p38 MAP kinase pathway because the specific and potent inhibitors of an upstream p38 kinase, SB203580 inhibited the induction of TLR4 protein. This signaling pathway of p38 was further confirmed by the finding that p38 siRNA inhibited the induction of TLR4 protein by cyclic stretch. The NF-κB binding activity and TLR4 promoter activity induced by cyclic stretch were attenuated by p38 inhibitor, indicating p38 MAP kinase plays an important role in the regulation of TLR4 expression by cyclic stretch in cardiomyocytes. TLR4 mediates through a phosphoinositide 3-kinase dependent pathway, not through p38 MAP kinase to protect against myocardial ischemia/reperfusion injury [21]. Recently, Bruns et al. have demonstrated that TLR4 inactivation resulted in an attenuation of several responses, including p38 MAP kinase phosphorylation and NF-κB nuclear translocation, which resulted in preventing burn-induced myocardial contractile dysfunction [22]. These data indicate that different signaling pathways may mediate TLR4 expression on cardiomyocytes in different stress states.

Mechanical stretch may induce secretion or synthesis of bioactive molecules from cardiomyocytes [23]. Wolf et al. have reported that AngII up-regulates TLR4 on mesanginal cells [24]. However, the link between TLR4 and AngII in cardiomyocytes has yet not been reported. TNF-α and interleukin-1 have been linked to TLR4 to mediate postischemic cardiac dysfunction [2]. In the present study, we have demonstrated that stretched cardiomyocytes secrete TNF-α and AngII. Exogenous addition of TNF-α or AngII increased TLR4 expression in cardiomyocytes. TNF-α monoclonal antibody and TNF-α receptor antibody blocked the increases of TLR4 protein induced by cyclic stretch. Losartan, an antagonist of AngII receptor attenuated the TLR4 protein expression induced by cyclic stretch and TNF-α, indicating that TNF-α works before AngII to induce TLR expression and AngII works through AngII receptor to enhance TLR4 expression in cardiomyocytes. This was confirmed by the finding that TNF-α increased AngII secretion and TNF-α antibody blocked the secretion of AngII secretion. These results provide the first evidence for TNF-α and AngII mediating cyclic stretch-induced expression of TLR4 in cardiomyocytes. These results further confirmed the autocrine or paracrine production of cardiomyocytes in response to cyclic stretch.

TLRs are a family of molecules that play a critical role in innate immunity. Among TLRs, TLR4, the receptor for lipopolysaccharide, is the most frequently observed and best-characterized receptor. In addition to playing a role in heart failure and myocardial ischemia/reperfusion injury, TLR4 has been found to play a role in neointimal formation, atherosclerosis [25] and stroke [26]. NF-κB is a critical transcription factor in TLRs-mediated signaling pathways [27]. In this study, we demonstrated that cyclic stretch stimulation of NF-κB -DNA binding activity required at least phosphorylation of the p38 since p38 inhibitor abolished the NF-κB binding activity. The phosphorylation of NF-κB protein was enhanced by cyclic stretch and was attenuated by p38 inhibitor. We further demonstrated that cyclic stretch increased TLR4 promoter activity and the binding site of NF-κB in the TLR4 promoter is essential for the transcriptional regulation. Our data indicate that NF-κB plays an important role in the regulation of TLR4 by cyclic stretch in cardiomyocytes.

## Conclusions

Our study reports for the first time that cyclic stretch enhances TLR4 expression in cultured rat cardiomyocytes. The stretch-induced TLR4 is mediated through TNF-α, AngII, p38 kinase and NF-κB pathway. TLR4 increases adhesion of monocytes to stretched myocytes. The TLR4 induced by cyclic stretch may contribute to the host defense of cardiomyocytes under hemodynamic overload.

## List of abbreviations used

Ang II: angiotensin II; EMSA: electrophoretic mobility shift assay; NF-κB: nuclear factor-kappa B; siRNA: small interfering RNA; TLR: toll-like receptor; TNF-α: tumor necrosis factor-α.

## Competing interests

The authors declare that they have no competing interests.

## Authors' contributions

K-GS has participated in the design of the study and drafted the manuscript. B-WW has made substantial contributions to conception and design, or acquisition of data, or analysis and interpretation of data. C-ML has made substantial contributions to conception and design, or acquisition of data, or analysis and interpretation of data. HC has given final approval of the version to be published.

## Supplementary Material

Additional file 1 **Supplementary figure**. Cyclic stretch increases angiotensin II receptor (Ang II-R) and tumor necrosis factor-α receptor (TNF-α-R) protein expression in cardiomyocytes. (A) Representative Western blots for Ang II-R and TNF-α-R in cardiomyocytes subjected to cyclic stretch by 20% for various periods of time. (B) Quantitative analysis of Ang II-R and TNF-α-R protein levels. The values from stretched cardiomyocytes have been normalized to values in control cells and the data from 4 independent experiments. *P < 0.001 vs. control. **P < 0.05 vs. control. (n = 4 per group). Click here for file
