# Supplementary material for: Cyclic stretch enhances the expression of Toll-like Receptor 4 gene in cultured cardiomyocytes via p38 MAP kinase and NF-κB pathway
Source: J Biomed Sci. 2010 Mar 5;17(1):15. doi: 10.1186/1423-0127-17-15 (PMC2844375; doi:10.1186/1423-0127-17-15)
Supplement: Additional file 1 — Supplementary figure. Cyclic stretch increases angiotensin II receptor (Ang II-R) and tumor necrosis factor-α receptor (TNF-α-R) protein expression in cardiomyocytes. (A) Representative Western blots for Ang II-R and TNF-α-R in cardiomyocytes subjected to cyclic stretch by 20% for various periods of time. (B) Quantitative analysis of Ang II-R and TNF-α-R protein levels. The values from stretched cardiomyocytes have been normalized to values in control cells and the data from 4 independent experiments. *P < 0.001 vs. control. **P < 0.05 vs. control. (n = 4 per group). [file 1423-0127-17-15-S1.PPT]

## Slide 1
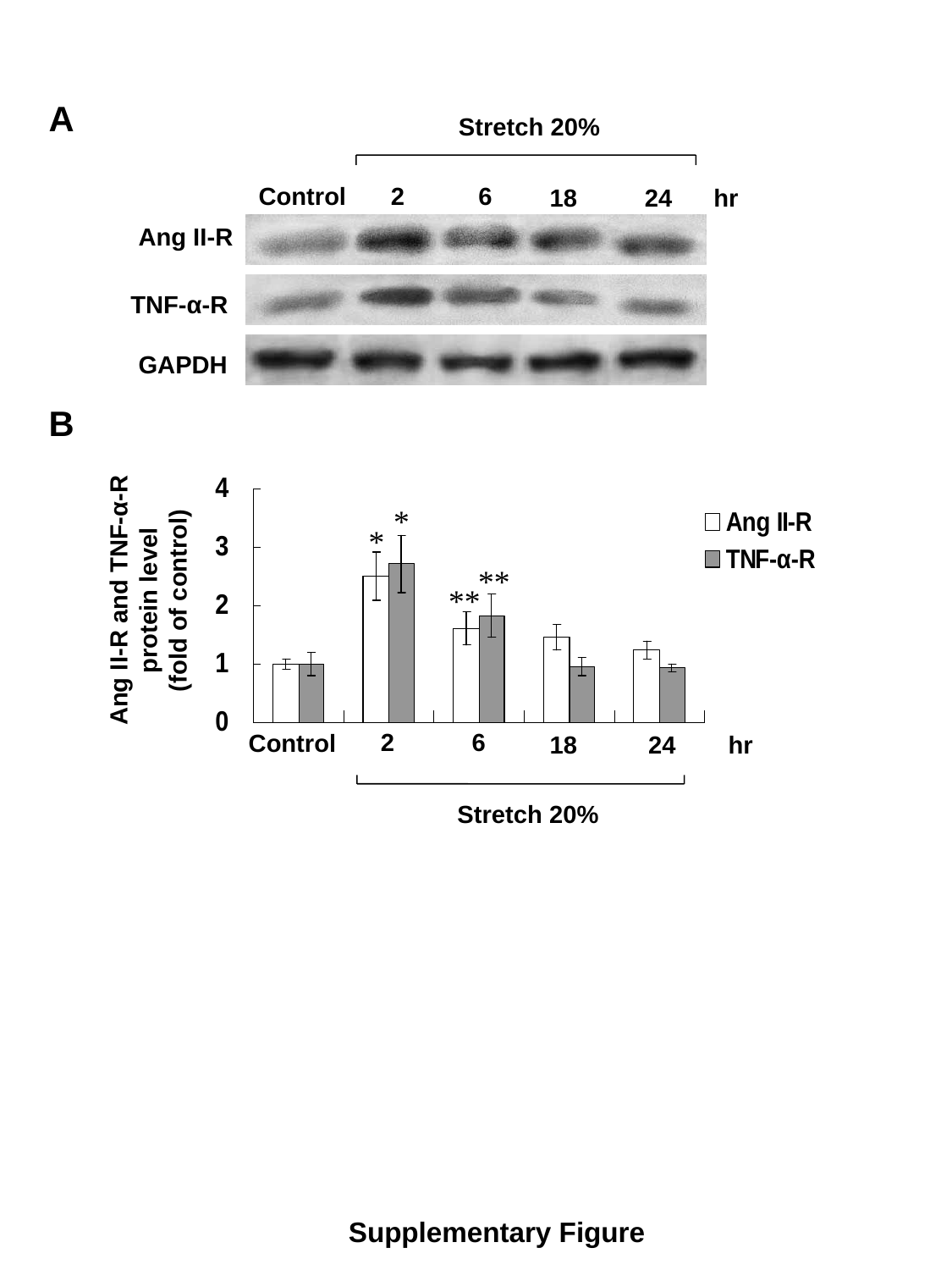

A
Stretch 20%
2
6
Control
18
24
 hr
Ang II-R
TNF-α-R
GAPDH
B
*
*
Ang II-R and TNF-α-R protein level
(fold of control)
**
**
2
6
Control
 hr
18
24
Stretch 20%
Supplementary Figure
